# Peptide mimetics of immunoglobulin A (IgA) and FcαRI block IgA‐induced human neutrophil activation and migration

**DOI:** 10.1002/eji.201646782

**Published:** 2017-09-06

**Authors:** Marieke H. Heineke, Lydia P.E. van der Steen, Rianne M. Korthouwer, J. Joris Hage, Johannes P.M. Langedijk, Joris J. Benschop, Jantine E. Bakema, Jerry W. Slootstra, Marjolein van Egmond

**Affiliations:** ^1^ Department of Molecular Cell Biology and Immunology VU University Medical Center Amsterdam The Netherlands; ^2^ Department of Plastic and Reconstructive Surgery Netherlands Cancer Institute‐Antoni van Leeuwenhoek Hospital Amsterdam The Netherlands; ^3^ Pepscan Therapeutics, Zuidersluisweg 2 Lelystad The Netherlands; ^4^ Department of Otolaryngology/Head‐Neck Surgery VU University Medical Center Amsterdam The Netherlands; ^5^ Department of Surgery VU University Medical Center Amsterdam The Netherlands

**Keywords:** Autoimmune blistering skin disease, CD89, Epitope mapping, Fc alpha receptor, Immunoglobulin A, Neutrophil, Peptide mimetic

## Abstract

The cross‐linking of the IgA Fc receptor (FcαRI) by IgA induces release of the chemoattractant LTB4, thereby recruiting neutrophils in a positive feedback loop. IgA autoantibodies of patients with autoimmune blistering skin diseases therefore induce massive recruitment of neutrophils, resulting in severe tissue damage. To interfere with neutrophil mobilization and reduce disease morbidity, we developed a panel of specific peptides mimicking either IgA or FcαRI sequences. CLIPS technology was used to stabilize three‐dimensional structures and to increase peptides’ half‐life. IgA and FcαRI peptides reduced phagocytosis of IgA‐coated beads, as well as IgA‐induced ROS production and neutrophil migration in in vitro and ex vivo (human skin) experiments. Since topical application would be the preferential route of administration, Cetomacrogol cream containing an IgA CLIPS peptide was developed. In the presence of a skin permeation enhancer, peptides in this cream were shown to penetrate the skin, while not diffusing systemically. Finally, epitope mapping was used to discover sequences important for binding between IgA and FcαRI. In conclusion, a cream containing IgA or FcαRI peptide mimetics, which block IgA‐induced neutrophil activation and migration in the skin may have therapeutic potential for patients with IgA‐mediated blistering skin diseases.

## Introduction

Autoimmune blistering skin diseases are characterized by autoantibodies against distinct structural desmosomal or hemidesmosomal proteins in the skin, which can lead to extremely itchy lesions or (sub)epidermal blisters 1, 2. Immunoglobulin A (IgA) pemphigus, linear IgA bullous disease (LABD) and dermatitis herpetiformis (DH) are blistering skin diseases characterized by aberrant deposits of IgA autoantibodies in the skin as well as dense inflammatory infiltrates that are dominated by neutrophils 2. IgA (auto)antibodies can activate FcαRI, a Fc receptor expressed on cells of the myeloid lineage including neutrophils, eosinophils, monocytes, and several macrophage subsets 3. Cross‐linking of FcαRI by IgA‐immune complexes initiates robust inflammatory responses, including superoxide production, release of cytokines, phagocytosis, antigen presentation and release of neutrophil extracellular traps 3. Additionally, we previously identified a novel pro‐inflammatory role for IgA, as cross‐linking of FcαRI by IgA‐antigen complexes led to neutrophil migration 4. This is beneficial during bacterial infections, as a self‐containing positive migration feedback loop can be initiated by IgA‐opsonized bacteria, until clearance of invading pathogens by neutrophils has been achieved. However, abnormal accumulation of IgA‐autoantigen complexes in tissues may lead to continuous neutrophil recruitment and activation, resulting in serious tissue damage due to the persistent release of harmful inflammatory cytokines, reactive oxygen species and proteases by infiltrated cells 5. Additionally, we demonstrated that neutrophil migration and activation via FcαRI was responsible for tissue damage in patients with IgA blistering skin diseases 6.

Currently, the mainstay for treatment of IgA pemphigus, DH and LABD is general suppression of immune responses with dapsone in combination with topical and systemic corticosteroids, which can have substantial side effects such as cutaneous atrophy, osteoporosis, gastrointestinal disturbances or hematological abnormalities 7, 8. As the interaction between IgA and FcαRI initiates neutrophil activation, interfering with this binding is a more specific therapy than the currently used immunosuppressants, thereby minimalizing side effects. The use of specific monoclonal antibodies (mAbs) as therapeutic tools to treat autoimmune diseases has increased dramatically in the last decade 9, 10, 11. We previously demonstrated that anti‐FcαRI mAbs inhibited IgA‐induced migration 4, 6. However, as inflammation in blistering diseases occurs within the skin, a topical applied therapy is desirable, and penetration of mAbs into the skin is likely negligible due to their large size. Successful delivery of peptides through the epidermis has already been demonstrated, thereby inhibiting IgG‐mediated blistering skin disease in mice 12. Therefore, peptide mimetics that block IgA–FcαRI interactions and which are small enough to pass the epidermis may represent good candidates to treat patients with IgA‐mediated blistering diseases.

The structures of and interaction sites between FcαRI and IgA have been characterized. FcαRI consists of two extracellular immunoglobulin‐like domains (EC1 and EC2), a transmembrane region, and a short cytoplasmic tail. The two extracellular domains are folded with an angle of approximately 90° to each other 13. The binding site of IgA for FcαRI lies at the interface of the Cα2 and Cα3 domains and comprises a central hydrophobic interface involving residues Leu257 and Leu258 on a loop at the “lower” end of Cα2, and Leu441, Ala442, and Phe443 on Cα3 13, 14, 15. Met433, Arg382, and some surrounding charged residues also contribute to the binding. The interaction site on FcαRI resides in the EC1 domain. Especially residues Tyr35, Leu54, Phe56, Gly84, His85 and Lys55 of FcαRI form the hydrophobic core of the interaction, with contributions from a number of surrounding charged residues 13, 16, 17. In this paper, two strategies were employed to obtain peptide mimetics which interfere with IgA‐FcαRI interaction. First, peptides were designed based on the known interaction sites between FcαRI and IgA, and second, epitope mapping studies revealed possible new candidates. Both linear and cyclic CLIPS peptides could block IgA‐induced neutrophil migration and several mimetics are therefore prospective candidates to treat patients with IgA blistering skin diseases.

## Results

### Linear and cyclic peptide mimetics inhibit ligand binding to FcαRI

**Figure 1:**
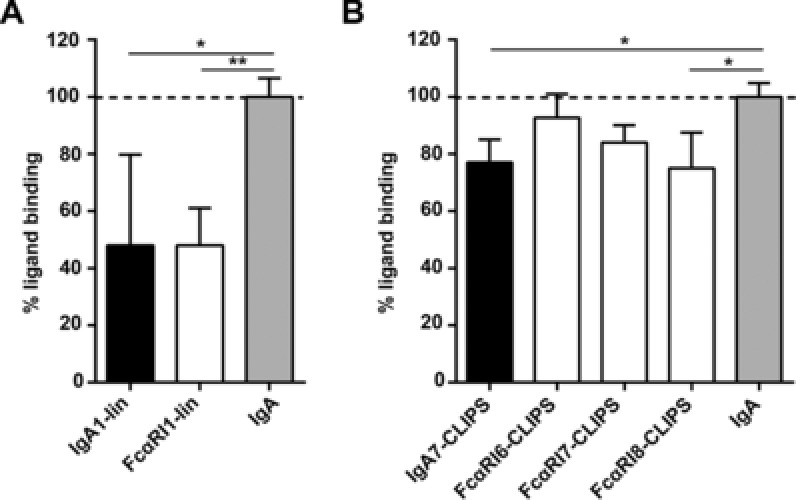
Peptide mimetics inhibit IgA binding to FcαRI. Percentage of ligand binding between IgA and FcαRI (on neutrophils), either in the presence or absence of peptide mimetics. Fluorescently labelled human neutrophils, isolated from blood, were added to IgA‐coated plates, and the number of neutrophils attaching to the plate was measured with a fluorimeter. IgA binding to FcαRI was normalized to 100% (indicated with dotted line). Neutrophils or plates were pre‐incubated with either linear peptides (A) or cyclic CLIPS‐peptides (B) mimicking FcαRI‐sequences (white bars) or IgA‐sequences (black bars). Data are representative of three independent experiments, performed in triplicates. Mean is shown, error bars refer to SD (*n* = 3). Statistical analysis: ANOVA*p< 0.05, **p < 0.01.

To determine whether peptide mimetics are able to block IgA binding to FcαRI, a panel of different soluble peptides was created based on described interaction sites of FcαRI and IgA (Supporting Information Fig. 1) 13, 14, 16, 17. Peptides varied in composition of amino acid residues, size (7‐ to 18‐mers) and level of constraint (linear or cyclic peptides, Table 1 and 2). Linear peptides mimicking either FcαRI (FcαRI1‐lin) or IgA (IgA1‐lin) demonstrated blocking of neutrophil binding to IgA‐coated wells with approximately 50% (Fig. 1A). CLIPS technology was used to cyclize the peptide and to thereby increase peptide half‐life and stability 18, 19. Cyclic peptides were less effective in blocking binding as they showed blocking capacities between 5 and 25% (Fig. 1B).

**Table 1:** FcαRI‐peptide sequences

	FcαRI‐peptide sequences^a^	Abbreviation
Original FcαRI sequence	GRYQCQYRIGHYRFRYSD	
Linear peptides	FcαRI1‐lin‐GRYQAQYRIGHYRFRYSD FcαRI2‐lin‐GRYQCQYRIGHYRFRYSD	FcαRI1‐lin FcαRI2‐lin
Cyclic peptides: CLIPS‐variant	FcαRI3‐CLIPS‐CHYRFRC FcαRI4‐CLIPS‐CRIGHYRFRC FcαRI5‐CLIPS‐YQACHYRFRC FcαRI6‐CLIPS‐RYQAQCRIGHYRFC FcαRI7‐CLIPS‐GRYQCQYRIGHYRFRYCD FcαRI8‐CLIPS‐GRYQACYRIGHYRFRCSD FcαRI9‐CLIPS‐GRYQAQCRIGHYRFCYSD	FcαRI3‐CLIPS FcαRI4‐CLIPS FcαRI5‐CLIPS FcαRI6‐CLIPS FcαRI7‐CLIPS FcαRI8‐CLIPS FcαRI9‐CLIPS
Cyclic peptides: oxidated‐variant	FcαRI6‐ox‐RYQAQCRIGHYRFC FcαRI7‐ox‐GRYQCQYRIGHYRFRYCD FcαRI8‐ox‐GRYQACYRIGHYRFRCSD FcαRI9‐ox‐GRYQAQCRIGHYRFCYSD	FcαRI6‐ox FcαRI7‐ox FcαRI8‐ox FcαRI9‐ox

a Underlined amino‐acids: difference compared to original sequence.

**Table 2:** IgA‐peptide sequences

	IgA‐peptide sequences^a^	Abbreviation
Original IgA sequence	SCMVGHEALPLAFTQKT	
Linear peptide	IgA1‐lin‐SSMVGHEALPLAFTQKT	IgA1‐lin
Cyclic peptides: CLIPS‐variant	IgA2‐CLIPS‐CEALPLAFTCKT IgA3‐CLIPS‐SCEALPLAFTCKT IgA4‐CLIPS‐SCMVGHEALPLAFTQCT IgA5‐CLIPS‐CSMVGHEALPLAFTQKC IgA6‐CLIPS‐SSMCGHEALPLAFCQKT IgA7‐CLIPS‐SSCVGHEALPLAFTCKT	IgA2‐CLIPS IgA3‐CLIPS IgA4‐CLIPS IgA5‐CLIPS IgA6‐CLIPS IgA7‐CLIPS
Cyclic peptides: oxidated‐variant	IgA6‐ox‐SSMCGHEALPLAFCQKT IgA7‐ox‐SSCVGHEALPLAFTCKT	IgA6‐ox IgA7‐ox

a Underlined amino‐acids: difference compared to original sequence.

### Peptides reduce IgA‐induced effector functions of neutrophils

**Figure 2:**
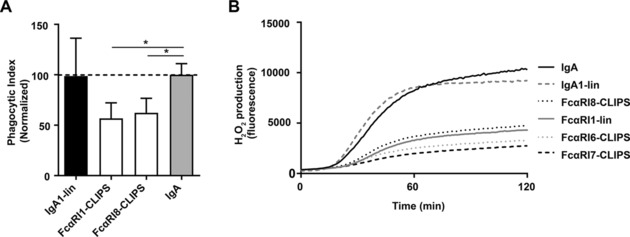
Peptide mimetics inhibit IgA‐induced ROS production and phagocytosis of IgA‐bead**s**. (A) Neutrophils, isolated from blood, were incubated with fluorescent IgA‐coated beads, in the presence or absence of peptide mimetics. Phagocytic index was calculated as the percentage of neutrophils that had phagocytosed beads, multiplied by the geometric mean of fluorescent cells. IgA binding to FcαRI was normalized to 100% (indicated with dotted line). Data are pooled from three independent experiments, performed in triplicates. Mean ± SD is shown (*n* = 5). Statistical analysis: ANOVA *p< 0.05. (B) Production of H_2_O_2_ as after adding neutrophils to IgA‐coated plates, as determined with an Amplex Red hydrogen peroxide assay. Neutrophils or plates were pre‐incubated with indicated peptides. Data are representative of three independent experiments, performed in triplicates. Mean of one representative experiment is shown (*n* = 3).

Subsequently, it was investigated if blocking IgA‐FcαRI interactions with IgA or FcαRI peptide mimetics inhibited IgA‐induced effector functions of neutrophils. FcαRI1‐lin and FcαRI8‐CLIPS were able to block phagocytosis of IgA‐coated beads with approximately 50% (Fig. 2A). Furthermore, H_2_O_2_ production was measured with an Amplex Red assay by adding neutrophils to IgA‐coated wells. Several peptides were capable of blocking H_2_O_2_ production (Fig. 2B).

### IgA‐induced neutrophil migration is reduced in the presence of peptide mimetics in vitro and ex vivo

**Figure 3:**
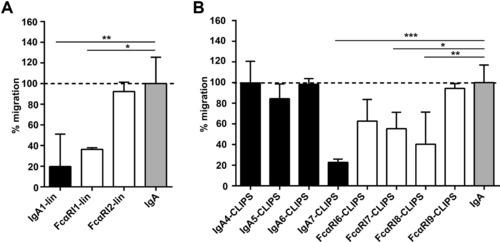
Peptide mimetics block IgA‐induced migration in vitro. Percentage of migration of fluorescently labelled neutrophils to IgA‐coated beads, either in the presence or absence of peptide mimetics. The number of migrated neutrophils was determined with a fluorimeter. Neutrophil migration to IgA was normalized to 100% (dotted line). Neutrophils or beads were pre‐incubated with (A) linear or (B) cyclic peptides mimicking FcαRI‐sequences (white bars) or IgA‐sequences (black bars). Data are representative of three independent experiments, performed in triplicates. Mean ± SD is shown. Statistical analysis: ANOVA *p< 0.05, **p < 0.01.

Next, we investigated whether peptide mimetics inhibit IgA‐induced neutrophil migration. Linear peptides FcαRI1‐lin and IgA1‐lin blocked neutrophil migration to IgA‐coated beads with 60–80% (Fig. 3A). CLIPS‐peptides blocked migration of neutrophils between 0 and 80% (Fig. 3B). Additionally, we tested oxidated peptide variants and smaller CLIPS‐peptide variants (7‐13 amino‐acids), but these peptides did not block IgA‐induced neutrophil migration (Supporting Information Fig. 2). Importantly, neither linear nor CLIPS peptides had an effect on IL‐8 induced chemotaxis (Supporting Information Fig. 3).

**Figure 4:**
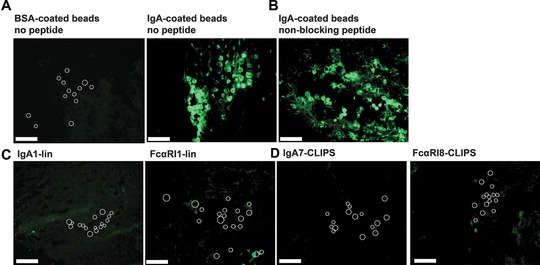
Peptide mimetics block neutrophil migration and penetrate the dermis in an ex vivo human skin model. Migration of green‐fluorescent neutrophils to BSA‐ (left panel) or IgA‐ (right panel) coated beads (indicated with circles) in the dermis of ex vivo skin explants (A). Migration of green‐fluorescent neutrophils to IgA‐coated beads (indicated with circles) after pre‐incubation with non‐blocking peptide FcαRI9‐ox (B), linear peptides (C) or cyclic CLIPS‐peptides (D). Images are representative of three independent experiments, performed in duplicate. Scale bar = 250 μm. Images are 10× magnified.

To mimic blocking of neutrophil migration toward aberrant IgA‐antigen complexes in the skin, an ex vivo migration assay was established. Full thickness human skin grafts were injected with IgA‐coated beads (or BSA‐coated beads as control) and incubated for 24 h with fluorescently labeled neutrophils in the absence or presence of peptides with the best blocking capacities as demonstrated in previous in vitro migration experiments. No influx toward BSA‐coated beads was observed, whereas massive influx of neutrophils toward the injected IgA‐coated beads was observed (Fig 4A). The non‐blocking peptide mimetic FcαRI9‐ox did not inhibit IgA‐induced migration (Fig. 4B). However, when IgA‐peptide IgA1‐lin or FcαRI‐peptide FcαRI1‐lin were added, neutrophil migration to IgA‐beads was completely blocked (Fig. 4C). Furthermore, cyclic peptides IgA7‐CLIPS and FcαRI8‐CLIPS fully abrogated neutrophil migration as well (Fig. 4D).

### Penetration of peptide mimetic IgA7‐CLIPS in human skin

**Figure 5:**
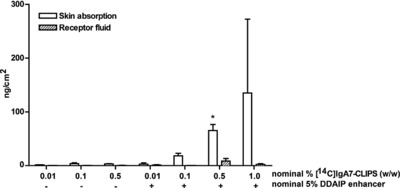
Penetration of peptide mimetic IgA7‐CLIPS in human skin. Penetration of radioactive labeled peptide mimetic ([^14^C]IgA7‐CLIPS) in ointment in ex vivo skin (white bars) and receptor fluid (striped bars). Recovery of peptide after 24‐h exposure is presented as ng/cm^2^ skin. Cream was applied with (+) or without (‐) nominal 5% permeation enhancer dodecyl‐2‐N, N‐dimethylaminopropionate (DDAIP). Data are shown as mean ±SD from a single experiment, performed in duplicates.

Ultimately, we aim to develop a topical therapy for patients with chronic IgA‐blistering diseases, which requires an ointment containing peptides which can penetrate into the skin. Therefore, we next analysed the potential dermal delivery of one of the cyclic peptides with the best blocking capacities demonstrated in in vitro and ex vivo migration experiments. An ointment containing radioactive labelled IgA‐peptides ([^14^C]IgA7‐CLIPS) was applied to skin explants and penetration of the peptides was determined. Without a skin permeation enhancer, minimal penetration of peptides into skin was observed. However, in presence of the enhancer DDAIP, a dose dependent increase of the amount of penetrated peptide mimetic was observed (Fig. 5). Moreover, the concentration of [^14^C]IgA7‐CLIPS in receptor fluid, which is a measure of systemic delivery, was negligible. In conclusion, topical application of a cream containing an enhancer resulted in local delivery of peptide mimetics.

### Epitope mapping studies reveal sequences important for binding between IgA and FcαRI

**Figure 6:**
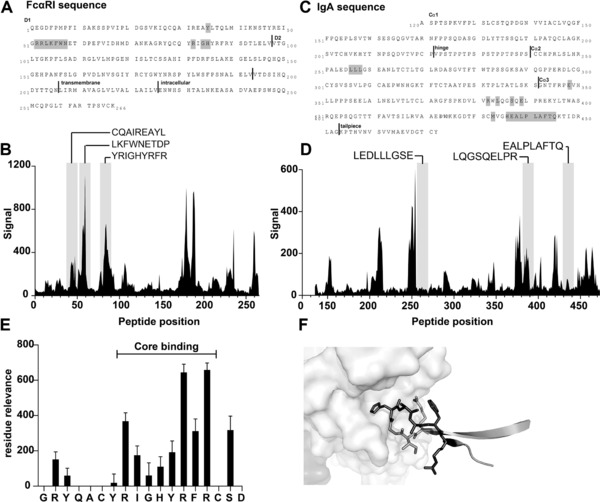
Epitope mapping studies reveal binding sequences of FcαRI and IgA. (A) Amino‐acid sequence of FcαRI. Vertical lines indicate different domains. Amino‐acids that are involved in binding with IgA are highlighted in gray. NCBI accession number for FcαRI is P24071 and based on Maliszewski *et al*.^20^ (B) IgA binding to FcαRI peptide library. Peptide positions indicate the sequence position of the N‐terminal residue of each 15‐mer peptide. Signal is the observed quantitative binding of the screened sample to the peptide. Gray regions are sequence areas predicted to be involved in the IgA‐FcαRI interaction. (C) Amino‐acid sequence of the constant regions of IgA. Gray highlighted residues have been documented to be involved in binding to FcαRI. NCBI accession number for IgA is P01876 and based on Woof *et al*.^21^. The IgA sequence is numbered according to the commonly adopted scheme used for human myeloma IgA1 protein Bur.^22^ (D) Screening of soluble FcαRI against the IgA peptide library, visualized as in B. (E) In‐depth analysis of peptide GRYQACYRIGHYRFRCSD (FcαRI8‐CLIPS). Residues involved in core binding are indicated, as mutation of these residues resulted in loss of function (binding). Data are shown as mean ±SD from a single experiment, performed in duplicate. (F) Schematic model of interaction of the amino‐acids of the FcαRI‐peptide GRYQACYRIGHYRFRCSD (FcαRI8‐CLIPS) with IgA‐Fc. Residues YRIGHYRFR are highlighted as “stick” visualization.

Next to the generated peptides based on the known interaction sites of IgA and FcαRI, we used epitope mapping to identify potential novel peptides which block IgA‐FcαRI interactions. Possibly, this second strategy allows us to discover novel interacting sequences, which are not described in current literature. Screening of a FcαRI‐peptide library with IgA identified strongly binding peptides on 5 regions of FcαRI (Fig. 6A and B). These included all three regions known to be involved in binding IgA (CQAIREAYL, LKFWNETDP and YRIGHYRFR), and at least two regions unrelated to the known binding interface. In the reciprocal experiment, an IgA‐peptide library was screened with both soluble FcαRI and 293T cells that had been transfected with FcαRI, with both experiments yielding similar results (Fig. 6C and D). Some binding was observed for IgA peptides which covered two out of three loops forming the previously described binding interface between IgA and FcαRI (LQGSQELPR and EALPAFTQ). However, within the complete dataset these were not the strongest binding regions. For region LEDLLLGSE no binding was observed, although this region is documented to form part of the binding interface.

Next, we investigated the specific residues important for the binding between FcαRI‐mimetic GRYQACYRIGHYRFRCSD (FcαRI8‐CLIPS) and IgA. This peptide is a strong binder and covers a region of FcαRI known to be important for the interaction with IgA. The analysis was performed by synthesizing a full positional replacement library for this sequence. By screening this mutated library for binding to IgA, the core binding region, critical residues and segments of the peptide that may be targets for future binding optimization studies could be identified. From this analysis region RIGHYRFR emerged as the core binding region, in which R87 and R89 showed the strongest effect on binding (Fig. 6E). When the identified core region was plotted on the structure of FcαRI and IgA, functional overlap was observed (Fig. 6F). In summary, epitope mapping studies revealed several novel sequences important for binding between IgA and FcαRI. Additionally, the core binding region of a promising FcαRI mimetic was discovered. Further studies are necessary to investigate if these peptides can block the IgA‐FcαRI interaction and thereby reduce neutrophil infiltration during IgA‐mediated blistering skin diseases.

## Discussion

**Figure 7:**
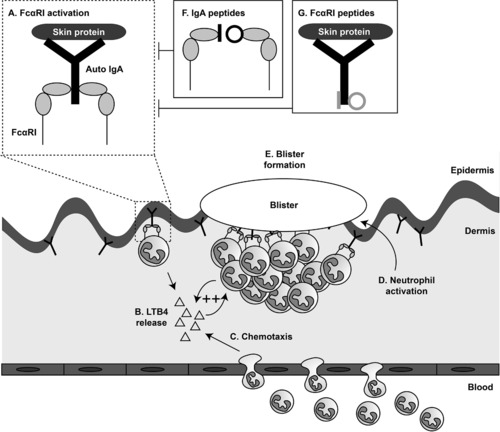
Working model: peptide mimetics prevent activation of neutrophils. IgA autoantibodies directed against skin epitopes activate FcαRI on neutrophils (A). This leads to release of LTB4 (B), initiating chemotaxis of neutrophils to the skin (C). These neutrophils get activated and also release LTB4, thereby initiating a positive feedback loop (arrows). Activated neutrophils release ROS and other toxic molecules (D), ultimately leading to tissue damage and blister formation (E). Linear and cyclic peptide mimetics of IgA (F) or FcαRI (G) in a topical ointment block the interaction between IgA‐FcαRI and thereby prevent neutrophil activation and migration. Of note, as example we depicted a sub‐epidermal blistering disease such as linear IgA bullous disease. However, IgA‐induced neutrophil activation and migration can also occur in epidermal blistering skin diseases such as IgA pemphigus, in which case neutrophil influx and blisters are located in the intraepidermal area (not shown).

In this paper a novel therapy for patients with IgA‐mediated chronic blistering skin diseases was explored. Patients with IgA‐mediated chronic blistering diseases suffer from severe itch and pain causing psychological, physical, social and economic problems 23. The current general immunosuppressive treatment consisting of dapsone and corticosteroids causes several undesirable side effects systemically 7, 8, and a more specific therapy is required. Since autoantibodies targeting skin proteins have been shown to be pathogenic in several mouse models 24, 25, 26, 27, 28, 29, 30, 31, 32, it is appealing to interfere with their binding or function. Removing autoantibody‐immune complexes by immunoadsorption or immunoapheresis is consequently an attractive strategy 33, 34, but this is an intensive treatment, since the patient's plasma needs to be filtered multiple times. Hence, we searched for an alternative. IgA (auto)antibodies can activate FcαRI with their Fc‐tail, resulting in neutrophil activation and migration, ultimately leading to tissue damage (Fig. 7). Therefore, regardless of the specific autoantigen to which auto‐IgA binds, it is attractive to interfere with the binding between the Fc‐tail of IgA and FcαRI in different IgA‐mediated diseases. Since the skin is (severely) damaged in these diseases, the preferential route of administration is topical. Although monoclonal antibodies have been fruitful to treat autoimmune diseases like rheumatoid arthritis and inflammatory bowel disease 10, 35, their molecular size prevents use in a topical ointment. The alternative approach to use peptides as protein mimics has been promising. Several investigations indicate that peptide mimetics can block protein‐protein interactions and attenuate immune responses 19, 36, 37, 38. By utilizing peptide mimetics, it is possible to specifically inhibit IgA‐mediated neutrophil activation and thereby interfere with pathogenicity at the site of inflammation. Patients with IgA‐mediated autoimmune diseases could therefore benefit of therapy containing peptide mimetics, which may result in reduced side effects.

Next to establishing that peptide mimetics successfully block IgA‐FcαRI interactions in in vitro and ex vivo experiments, a potential route of administration was investigated in this study. The use of microneedles (‘tattooing’) has been shown to be a very efficient approach for peptide vaccination 39, 40, 41. This array of microscopic needles is sufficiently long to penetrate the epidermis, but adequately small to prevent major skin injury or pain 42. Unfortunately, this approach is likely not feasible for patients with established blistering skin disease, as multiple administrations are required to block the influx of inflammatory cells in a skin which is already damaged. For these patients it is of utmost importance that a specific, topically applied therapy is developed. In this study we demonstrated that IgA‐peptides in ointment penetrated the epithelial barrier in a dose dependent manner when a skin permeation enhancer was added. Moreover, the concentration of peptide mimetics in the receptor fluid, which is a measure of systemic delivery, was negligible, supporting limited systemic exposure. Successful blocking of disease after topical delivery of peptides through the epidermis was previously demonstrated in vivo 12. Spindler et al. showed that when a peptide targeting desmoglein was applied topically in an ointment, IgG autoantibody‐mediated blistering skin in a pemphigus mouse model was abrogated 12. As such, an ointment containing peptides blocking IgA‐induced neutrophil migration represents a promising novel approach to specifically treat IgA‐mediated blistering skin diseases, which may decrease severe morbidity and improve quality of life for these patients.

## Materials and methods

### Peptide library synthesis

The described interaction sites of FcαRI and IgA (Supporting Information Fig. 1) were used to create a panel of soluble peptides, based on the amino‐acid sequence GRYQCQYRIGHYRFRYSD of FcαRI (Table 1) and on the amino‐acid sequence SCMVGHEALPLAFTQKT of IgA (Table 2).

### Synthesis of CLIPS‐peptides and SS‐peptides and peptide‐microarrays

Synthesis of CLIPS‐peptides, SS‐peptides (oxidated variants) and peptide microarrays was performed as described previously 19, 43.

### Pepscan‐based ELISA

Binding of soluble FcαRI or IgA Ab to plate‐bound peptides was tested with Pepscan‐based ELISA's, which were adapted according to the method previously described 19, 43, 44. The samples were washed to remove unbound fragments after each incubation step. The 455‐well credit‐card format polypropylene cards containing the covalently linked IgA‐peptides were incubated with blocking solution (4% horse serum, 5% ovalbumin (w/v) in PBS/1% Tween). Next, peptides were incubated with soluble FcαRI or 293T cells transfected with FcαRI and subsequently with mouse anti‐human FcαRI IgG mAb (1 μg/ml; BD, Franklin Lakes, NJ). Then, peptides were incubated with rabbit anti‐mouse IgG‐HRP (1/1000, Dako, P0212) for 1 h at RT. Alternatively, after blocking, the covalently linked FcαRI‐peptides were incubated with pooled human serum IgA (Cappel™, MP Biomedicals, Santa Ana, CA), and rabbit anti‐human IgA‐HRP (1 μg/mL, Dako, P0212) was added. The peroxidase substrate 2,2′‐azino‐di‐3‐ethylbenzthiazoline sulfonate (ABTS) and 2 μL of 3% H_2_O_2_ was added (1 h, RT). Colour development was measured, which was quantified with a charge coupled device (CCD)‐camera and an image processing system. Values mostly ranged from 0 to 3000, a log scale similar to 1–3 of a standard 96‐well plate ELISA‐reader.

### Epitope mapping

To identify the core binding region of peptide GRYQACYRIGHYRFRCSD (FcαRI8‐CLIPS), a full positional replacement library was created. A set of variants was designed in which one of the 17 single residues (C residues are excluded) was replaced by one of the 19 other available L‐amino acids, resulting in a dataset of 19 × 17 unique peptides. To identify the specific residues important for the interaction, this library was screened for binding to IgA as described above. The observed binding for each peptide variant was then analysed in comparison to the unmodified (“wt”) sequence. The amount of loss of binding observed for the variant, aggregated for each position in the sequence, is taken as a measure for the binding relevance for that residue. Within this library a relative value is then obtained for each position within the sequence which is used to determine the core binding region, critical residues and segments of the peptide that may be targets for future binding optimization studies.

### Generation of soluble FcαRI recombinant protein

To generate a recombinant HIS‐tagged soluble FcαRI protein, full length FcαRI inserted in pMG‐FcαRI‐IRES‐hFcR γ‐chain 45 was used as template. The extracellular part of FcαRI was amplified and cloned into pcDNA/v5‐his topo vector (Invitrogen, Life Technologies Europe BV, Bleiswijk, The Netherlands), making use of a Kozak sequence (CACCATG) at the end of the 5′ primer (gtc agc acg gcc acc atg gac cc) and 3′ primer (tgt cga gct agc tta gat caa gtt ctg cgt c). The vector construct was verified by sequence analysis. For protein expression, 293T cells were stable transfected with pcDNA3.1/v5‐sCD89 using FuGENE 6 reagent (Promega, Madison, USA), according to the manufacturer's recommendations and cultured under selection of hygromycin B. Protein expression of soluble FcαRI was verified by SDS‐PAGE and Western blotting using an anti‐FcαRI antibody (kindly provided by Prof. dr. C. van Kooten, LUMC, The Netherlands) 46. Furthermore, functionality of soluble FcαRI proteins was confirmed by FACS staining using soluble FcαRI protein as blocking agent for standard FcαRI staining on BAF3 transfected cells as described in Bracke *et al*. 47 with commercial anti‐FcαRI antibody (mouse anti‐human FcαRI‐PE; BD Pharmingen, Breda, The Netherlands).

### Isolation of human neutrophils

Neutrophils were isolated from heparinized peripheral human blood from healthy donors after informed consent was given, using standard Lymphoprep (Axis‐shield, Dundee, Scotland) density gradient centrifugation. Erythrocytes were removed by hypotonic lysis, after which neutrophils were resuspended in RPMI 1640 (Gibco BRL, Paisley, UK) supplemented with 10% heat‐inactivated fetal calf serum, L‐glutamine, and antibiotics. Neutrophils were labeled for 30 min at 37°C with 1 μmol/L calcein‐acetoxymethylester (Molecular Probes, Eugene, OR) for binding assays or with PKH‐67 (Sigma‐Aldrich, St. Louis, MO) for migration experiments according to the manufacturers’ instructions. Migration assays were performed in medium without fetal calf serum. Studies were performed according to the guidelines of the Medical Ethical Committee of VU University Medical Center, The Netherlands, in agreement with the Declaration of Helsinki.

### Preparation of immunoglobulin‐coated beads

N‐hydroxysuccinimide (NHS)‐activated sepharose beads (GE Healthcare, Uppsala, Sweden) were coated with pooled human serum IgA (300 μg/mL, Sigma‐Aldrich) according to the manufacturer's instructions.

### Ligand binding assay

Plates (Nunc‐ImmunoMaxiSorp™, Roskilde, Denmark) were coated with IgA, or BSA (as control) (10 μg/mL, 3 h, 37°C), washed and pre‐incubated with FcαRI‐peptides (1 μg/mL, 20 min, 4°C). Wells were subsequently incubated with calcein labeled neutrophils (2.5 × 10^5^ cells/well) for 20 min (37°C). Alternatively, calcein labeled neutrophils were pre‐incubated with IgA‐peptides (1 μg/mL, 20 min, 4°C) and subsequently added to IgA or BSA coated wells. Plates were thoroughly washed to remove non‐bound cells. Attached cells were lysed and fluorescence of supernatant was measured with a fluorimeter (485 nm excitation/520 nm emission filters; Fluostar Galaxy, BMG Labtechnologies, Offenburg, Germany), as measure of binding. All experiments were performed in triplicate.

### Phagocytosis assay

Phagocytosis assays with fluorescent latex beads were performed as described previously 48. IgA‐coated beads were added in ET ratio of 100 to neutrophils for 30 min at 37°C and fluorescence was measured with flow cytometry (FACS Calibur, BD Biosciences). Beads or neutrophils were pre‐incubated with peptides (1 μg/mL) for 20 min on ice. Phagocytic index was calculated as the percentage of cells that had phagocytosed, multiplied by the geometric mean of fluorescent cells.

### Amplex Red assay

Production of H_2_O_2_ was determined with Amplex™ Red hydrogen peroxide assays (Invitrogen, A‐12221). ELISA plates (Nunc) were coated with IgA (1 ug/mL). Neutrophils were incubated in 100 μL Hepes^+^ buffer (132 nM NaCl, 20 mM hepes, 6 mM KCl, 1 mM MgSO_4_·7H_2_O, 1.2 mM K_2_HPO_4_·3H_2_O, 1 mM CaCl_2_, 0.5% BSA, 1 mg/mL glucose). Neutrophils or plate were pre‐incubated with peptides (5 μg/mL) for 20 min on ice. Neutrophils were added to plates and Amplex Red reaction mix (200 μM Amplex red reagent and 4 U/mL horse radish peroxidase) was added. Fluorescence of the produced resorufin was measured every 1 min for 2 h at 37°C in a fluorimeter with an excitation of 550 nm and an emission of 590 nm.

### Neutrophil migration assays

In vitro migration assays were performed as previously described 4. All experiments were performed in triplo. Migration towards IL‐8 (30 ng/mL) was measured after pre‐incubating neutrophils with peptides (1 μg/mL) or anti‐IL‐8 blocking antibody (10 μg/mL, Pharmingen).

For ex vivo human skin migration assays, full thickness mammary skin grafts (epidermis and dermis) were placed in an ex vivo tissue incubation chamber with the dermis face up 6, 49. IgA‐coated beads were pre‐incubated with FcαRI‐peptides (1 μg/mL, 20 min, 4°C) and injected intracutaneously via the dermis. BSA‐coated beads were used for control. Next, PKH‐67 labeled neutrophils (4 × 10^6^ cells/well) were added on the dermis. Alternatively, IgA‐coated beads were injected intracutaneously and PKH‐67 labeled neutrophils (4 × 10^6^ cells/well) that were pre‐incubated with IgA‐peptides (1 μg/mL, 20 min, 4°C) were added onto the dermis. Cells were supplemented with IFN‐γ to prevent early apoptosis (300 units/mL; Boehringer Ingelheim, Ingelheim am Rhein, Germany). Skin was incubated overnight at 37°C, after which biopsies of the injected skin were taken and snap frozen. Cryosections of 6 μm were analysed with a Nikon eclipse e800 (Nikon instruments Europe BV, Amsterdam, The Netherlands).

### Ex vivo dermal absorption of [^14^C]IgA7‐CLIPS through human skin

The experiments were based on the protocol ‘OECD Environmental Health and Safety Publications, Series on Testing and Assessment no. 28. Guidance document for the conduct of skin absorption studies, Paris, March 2004’ and performed by Netherlands Organisation for Applied Scientific Research (TNO). In brief, human skin membranes were placed in Franz diffusion cells (PermaGear Inc., Riegelsville, PA). The skin surface temperature was kept at 32°C, at ambient humidity. The receptor fluid consisted of PBS containing 0.01% sodium azide. Radioactive labeled peptide mimetic [^14^C]IgA7‐CLIPS was made from Fmoc‐[1‐^14^C]glycine (Quotient Bioresearch (Radiochemicals) Ltd, Cardiff, UK). The material was purified by high performance liquid chromatography (HPLC) and the radiochemical purity was 99.3%. [^14^C]IgA7‐CLIPS was applied topically to the skin membranes in a Cetomacrogol‐cream (prepared at VU University Medical Center, Amsterdam, The Netherlands) in different concentrations and with or without skin permeation enhancer dodecyl‐2‐(N,N‐dimethylamino)propionate (DDAIP) 50. The concentration and homogeneity of [^14^C]IgA7‐CLIPS in the formulations was checked by taking additional weighed aliquots before and directly after dosing. After 24 h of application, the mass balance of the test substance was determined. Skin membranes were separated in epidermis and dermis using tweezers. Skin fractions were digested in a 1.5 M KOH solution with 20% ethanol for 24 h. Radioactivity in all samples was determined by liquid scintillation counting (LSC) on a Tri‐Carb 3100TR liquid scintillation counter using QuantaSmart™ software (PerkinElmer, Waltham, MA). All counts were converted to DPM (disintegration per minute) using tSIE/AEC (transformed Spectral Index of external standards coupled to Automatic Efficiency Correction).

### Statistical analysis

All analyses were performed using GraphPad Prism version 4.03 for Windows (GraphPad Software, San Diego, CA). Data are shown as mean ± standard deviation. Statistical differences were determined using two‐tailed unpaired Student's t‐test (comparing 2 groups) or ANOVA (comparing >2 groups). Significance was accepted when *p*< 0.05.

## Conflict of interest

The authors declare no financial or commercial conflict of interest.

**Abbreviations**
CLIPS: Chemical Linkage of Peptides onto Scaffolds
FcαRI: IgA Fc receptor I
IgA: immunoglobulin A
Ox: oxidated variant
mAbs: monoclonal antibodies

## Supporting information

Peer review correspondence

Supporting material
