# Supplementary material for: Peptide mimetics of immunoglobulin A (IgA) and FcαRI block IgA‐induced human neutrophil activation and migration
Source: Eur J Immunol. 2017 Sep 6;47(10):1835–45. doi: 10.1002/eji.201646782 (PMC5659136; doi:10.1002/eji.201646782)
Supplement: Supplementary file 2 — Supporting material [file EJI-47-1835-s002.pdf]

European Journal of Immunology

Supporting Information
for
DOI 10.1002/eji.201646782

Marieke H. Heineke, Lydia P.E. van der Steen, Rianne M. Korthouwer, J. Joris Hage, Johannes P.M. Langedijk, Joris J. Benschop, Jantine E. Bakema, Jerry W. Slootstra and Marjolein van Egmond

Peptide mimetics of immunoglobulin A (IgA) and FcαRI block IgA-induced human neutrophil activation and migration

© 2017 The Authors. *European Journal of Immunology* published by WILEY-VCH Verlag GmbH & Co. KGaA, Weinheim.

Supporting Information Figure 1

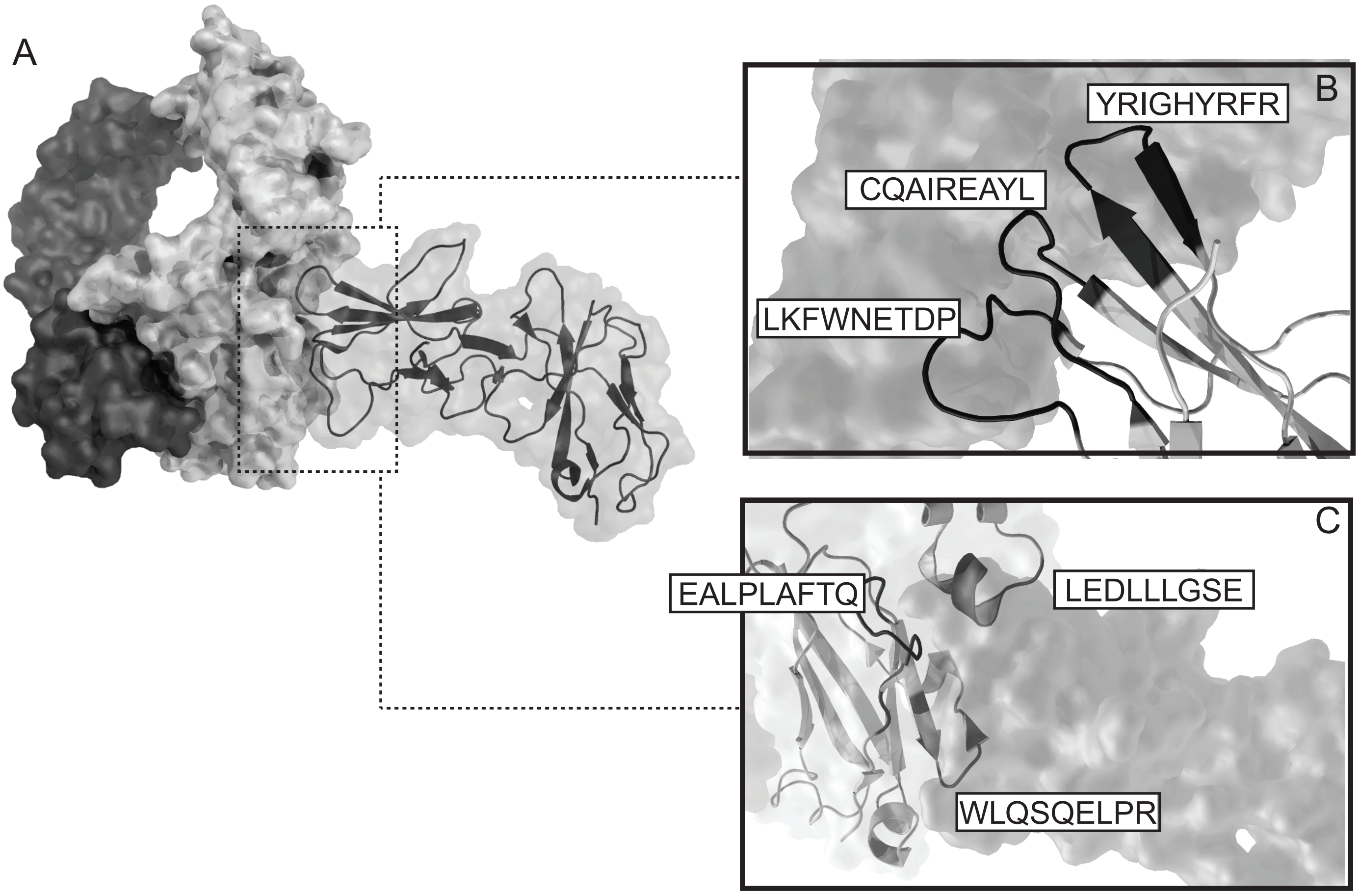

Supporting Figure 1. Model of interaction sites of IgA and FcaRI
(A) Model of the structure of the complex of two IgA Fc heavy chains, interacting with two extra-cellular domains of FcαRI. (Two molecules of FcαRI form a complex with one IgA molecule).
(B and C) Magnification of position of FcαRI sequences (B) and IgA sequences (C) involved in binding.

Supporting Information Figure 3

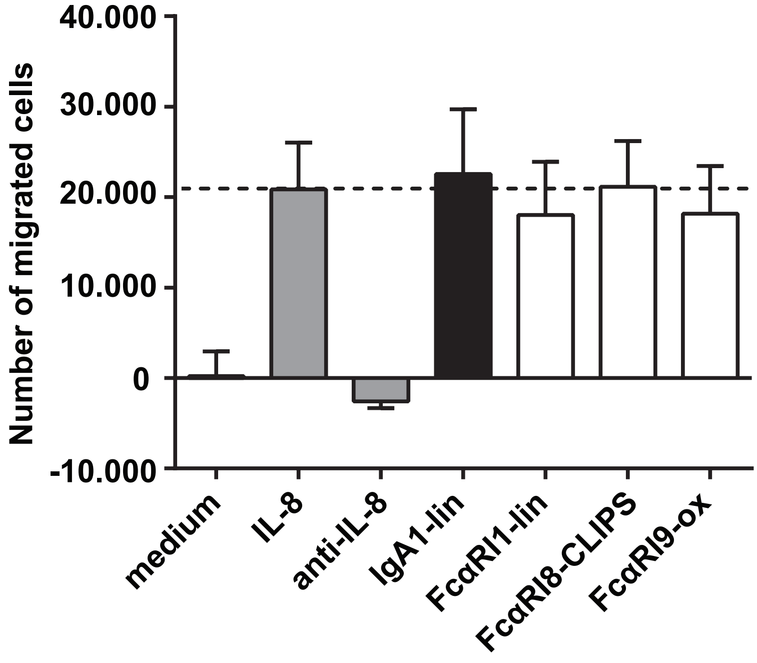

Supporting Figure 3. Peptide mimetics do not block IL-8 induced chemotaxis
Number of fluorescently labelled neutrophils which migrated to IL-8 in a chemotaxis chamber, either in the presence or absence of peptide mimetics. The number of migrated neutrophils was determined with a fluorimeter. Neutrophil migration to IL-8 is indicated with a dotted line. Medium and blocking IL-8 antibody (anti-IL-8) were used as controls. Experiments were performed three times in triplo. Mean ± SD of one representative experiments in triplo is shown. Statistical analysis: ANOVA

Supporting Information Figure 2

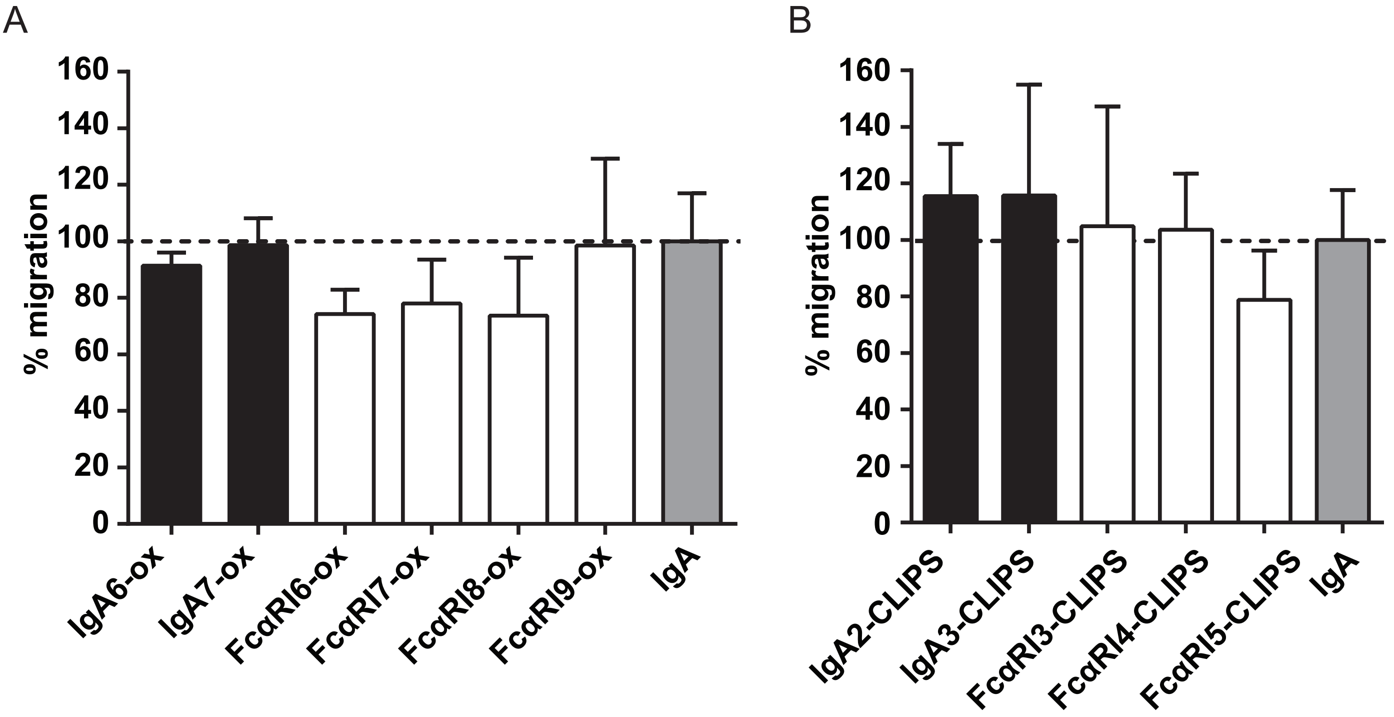

Supporting Figure 2. Oxidated or small peptide mimetics do not block IgA-induced migration *in vitro*

Percentage of migration of fluorescently labelled neutrophil to IgA-coated beads, either in the presence or absence of peptide mimetics. The number of migrated neutrophils was determined with a fluorimeter. Neutrophil migration to IgA was normalized to 100% (dotted line). Neutrophils or beads were pre-incubated with (A) oxidated or (B) small cyclic peptides mimicking FcαRI-sequences (white bars) or IgA-sequences (black bars). Data are representative of 3 independent experiments, performed in triplicates. Mean ± SD of one representative experiment is shown. Statistical analysis: ANOVA
