# Supplementary material for: Peptide mimetics of immunoglobulin A (IgA) and FcαRI block IgA‐induced human neutrophil activation and migration
Source: Eur J Immunol. 2017 Sep 6;47(10):1835–45. doi: 10.1002/eji.201646782 (PMC5659136; doi:10.1002/eji.201646782)
Supplement: Supplementary file 1 — Peer review correspondence [file EJI-47-1835-s001.pdf]

European Journal of Immunology

Peptide mimetics of immunoglobulin A (IgA) and FcαRI block IgA-induced neutrophil activation and migration

Marieke Heineke; Lydia van der Steen; Rianne Korthouwer; J. Joris Hage; Johannes Langedijk; Joris Benschop; Jantine Bakema; Jerry Slootstra; Marjolein van Egmond

Correspondence: Dr. van Egmond, Marjolein VU University Medical Center - Department of Molecular Cell Biology and Immunology, Amsterdam, Noord-Holland; Netherlands

---

Review Timeline:	Submission date:	27-Oct-2016
	First Editorial decision:	24-Nov-2016
	Revision received:	06-Jun-2017
	Accepted:	20-Jul-2017

Handling Executive Committee member: Prof. Iain McInnes

Please note that the correspondence below does not include the standard editorial instructions regarding preparation and submission of revised manuscripts, only the scientific revisions requested and addressed.

First Editorial Decision
24-Nov-2016

Dear Dr. van Egmond,

Manuscript ID eji.201646782 entitled "Peptide mimetics of immunoglobulin A (IgA) and FcαRI block IgA-induced neutrophil migration" which you submitted to the European Journal of Immunology has been reviewed. The comments of the referee(s) are included at the bottom of this letter.

A revised version of your manuscript that takes into account the comments of the referees will be reconsidered for publication. Should you disagree with any of the referees'concerns, you should address this in your point-by-point response and provide solid scientific reasons for why you will not make the requested changes.

You should also pay close attention to the editorial comments included below. **In particular, please edit your figure legends to follow Journal standards as outlined in the editorial comments. Failure to do this

will result in delays in the re-review process.**

Please note that submitting a revision of your manuscript does not guarantee eventual acceptance, and that your revision will be re-reviewed by the referees before a decision is rendered.

If the revision of the paper is expected to take more than three months, please inform the editorial office. Revisions taking longer than six months may be assessed by new referees to ensure the relevance and timeliness of the data.

Once again, thank you for submitting your manuscript to European Journal of Immunology and we look forward to receiving your revision.

Yours sincerely,
Nadja Bakocevic

On behalf of
Prof. Iain McInnes

Dr. Nadja Bakocevic
Editorial Office
European Journal of Immunology
e-mail: ejied@wiley.com
www.eji-journal.eu

****

Reviewer: 1

Comments to the Author
The comments to the authors:

In this study, to develop a novel therapy for IgA mediated autoimmune bullous skin diseases, the authors determined the inhibitory effects of various peptide mimetics of both IgA and FcαRI on IgA-related binding and migration of neutrophils by various methods. The results indicated that both linear and circular types of peptide mimetics of both IgA and Fcï• ¡RI could inhibit both binding and migration of neutrophils in either in vitro and ex vivo assays. Skin explants study showed that the peptides can penetrate into the skin, indicating that the peptide mimetics therapy is suitable for topical treatment. In addition, by using

epitope mapping methods, the authors also revealed possible new candidates for the peptide mimetics therapy for both IgA and FcαRI.

This study provides us with various novel insights into developing a novel therapy for IgA-mediated diseases, not only in the skin but also in other tissues. All experiments and statistical analyses have been well performed. Conclusions are adequate. English is well written.

Limitation of this study is that experiments directly related to skin diseases are only ex vivo skin explants cultures for neutrophil migration and penetration study of radio-labeled peptides, although the authors repeatedly emphasize the effectiveness of the peptide mimetics therapy in IgA-related autoimmune bullous skin diseases.

In addition, I have several comments and concerns, which described below.

(1) Although the authors suggest that the peptide mimetics therapy can substitute current therapy using DDS (dapsone), other sulphones or macrolides, the authors do not describe about it in details. For better understanding of the readers, the authors should mention how the therapeutic mechanism of peptide mimetics therapy is different from that in DDS therapy, which of the two therapies are superior in particular disease or patients, and how the practitioners (dermatologists) select one the two therapies in each patient in the introduction or discussion section.

(2) Although the aim of this study was to develop a new therapy for IgA-related autoimmune bullous skin diseases, there are no description for the relation of the therapy to autoantibodies to various skin component proteins, which are critical for development of skin lesions. Therefore, the authors should mention about their speculations how the autoantibodies and specific epitopes on the autoantigens may relate to efficacy of the peptide mimetics therapy in either introduction or discussion section.

(3) The term ‘œepitope’• usually means targeting region on antigen for antibody binding. Therefore, the term ‘œepitope mapping study’• for detection of regions suitable of the peptide mimetics therapy sounds strange to me. Therefore, to avoid misunderstanding of the readers, the authors may change the term ‘œepitope mapping study’• to more suitable term.

(4) In the Figures 1 and 2, the authors show the effectiveness of various peptides on inhibition of binding and migration of neutrophils as % blocking rates. However, such figures usually show the rate of binding or migration against the value of non-treated controls. Thus, the figures may look to show opposite results at a glance, and may cause confusion of the readers. Therefore, the authors may show the results as rate of binding and migration rather than % blocking rates of binding and migration.

(5) In the Figure 3, the orientation of section of skin explants, into which granulocytes with green fluorescence migrated, is unclear. The figures should be clarified by indicating the areas of the epidermis

and the dermis by arrows with help of experts of dermatological pathologists.

(6) The bottom panel of the Figure 5 is not clear, and probably wrong in some places. This event should be initiated by binding of IgA autoantibodies to skin autoantigens, which are missing. Blister formation should be induced, after migration and activation of neutrophils, although the figure shows the blister as the initial event. In addition, the sites in the epidermis for the blisters and neutrophilic accumulation are different between dermatitis herpetiformis/linear IgA bullous dermatosis (subepidermal area) and IgA pemphigus (or, intercellular IgA dermatosis) (intra-epidermal area). This should be also clearly shown. Positive feedback loop of neutrophil activation is not clearly depicted. The areas of the epidermis and the dermis are not indicated. The figures should be clarified by consulting to dermatologists. In addition, the possible mechanism for the induction of blister formation by activated neutrophils may be included into the figure.

Reviewer: 2

Comments to the Author
Heineke et al generated a panel of specific peptides mimicking the IgA and FcαRI sequences. They demonstrated that one of linear IgA peptides and a FcαRI peptide decrease IgA-mediated neutrophil migration. Using CLIPs technology to increase peptides' half-life, the authors established cyclic IgA peptide that completely blocked migration of neutrophils. Moreover, the authors have developed a cream containing CLIPS peptide and showed that this can penetrate to skin but not systemically open new therapeutic avenues for IgA-mediated blistering skin diseases.

Comments:
1. The authors should address the role of these cyclic peptides on other neutrophil functions such as reactive oxygen species production and phagocytosis.
2. They must also test the effect of theses peptides on neutrophil migration using IgA independent mechanisms such as MCP-1 and IL-8.

First Revision – authors' response

Reviewer: 1

Comments to the Author

In this study, to develop a novel therapy for IgA mediated autoimmune bullous skin diseases, the authors determined the inhibitory effects of various peptide mimetics of both IgA and FcαRI on IgA-related binding and migration of neutrophils by various methods. The results indicated that both linear and circular types of peptide mimetics of both IgA and FcαRI could inhibit both binding and migration of neutrophils in either in vitro and ex vivo assays. Skin explants study showed that the peptides can penetrate into the skin, indicating that the peptide mimetics therapy is suitable for topical treatment. In addition, by using epitope mapping methods, the authors also revealed possible new candidates for the peptide mimetics therapy for both IgA and FcαRI

This study provides us with various novel insights into developing a novel therapy for IgA-mediated diseases, not only in the skin but also in other tissues. All experiments and statistical analyses have been well performed. Conclusions are adequate. English is well written.

Limitation of this study is that experiments directly related to skin diseases are only ex vivo skin explants cultures for neutrophil migration and penetration study of radio-labeled peptides, although the authors repeatedly emphasize the effectiveness of the peptide mimetics therapy in IgA-related autoimmune bullous skin diseases.

In addition, I have several comments and concerns, which described below.

(1) Although the authors suggest that the peptide mimetics therapy can substitute current therapy using DDS (dapsone), other sulphones or macrolides, the authors do not describe about it in details. For better understanding of the readers, the authors should mention how the therapeutic mechanism of peptide mimetics therapy is different from that in DDS therapy, which of the two therapies are superior in particular disease or patients, and how the practitioners (dermatologists) select one the two therapies in each patient in the introduction or discussion section.

Response: Thank you for your valuable suggestion, we agree that this subject needs clarification. We have now described why peptide mimetic therapy is more specific than general suppression of autoimmune diseases in the introduction and discussion (highlighted in yellow, p.3 and p. 9).

(2) Although the aim of this study was to develop a new therapy for IgA-related autoimmune bullous skin diseases, there are no description for the relation of the therapy to autoantibodies to various skin component proteins, which are critical for development of skin lesions. Therefore, the authors should

mention about their speculations how the autoantibodies and specific epitopes on the autoantigens may relate to efficacy of the peptide mimetics therapy in either introduction or discussion section.

Response: The reviewer is correct that we do not discuss the specific epitopes of the different diseases in our paper, as binding to the epitopes is mediated by the Fab fragment of an antibody. However, the activation of neutrophils through Fc alpha receptor is mediated by the Fc tail of IgA, which occurs regardless of Fab binding to different epitopes. In our paper, we show we can block the IgA-Fc interaction with Fc alpha receptor. It is highly unlikely that the autoantigen has an influence on the peptide mimetic therapy. To make this a bit more clear, we changed the text (highlighted in yellow, p. 9).

(3) The term “epitope” usually means targeting region on antigen for antibody binding. Therefore, the term “epitope mapping study” for detection of regions suitable of the peptide mimetics therapy sounds strange to me. Therefore, to avoid misunderstanding of the readers, the authors may change the term “epitope mapping study” to more suitable term.

Response: Thank you for the suggestion, however the technique of identifying amino acid residues involved in binding is generally referred to by the term epitope mapping. As such, we cannot change this term.

(4) In the Figures 1 and 2, the authors show the effectiveness of various peptides on inhibition of binding and migration of neutrophils as % blocking rates. However, such figures usually show the rate of binding or migration against the value of non-treated controls. Thus, the figures may look to show opposite results at a glance, and may cause confusion of the readers. Therefore, the authors may show the results as rate of binding and migration rather than % blocking rates of binding and migration.

Response: Thank you for your valuable suggestion. We have changed the % blocking rates into % binding and % migration (Fig. 1 and 3), and subsequently changed the figure legends and material & methods.

(5) In the Figure 3, the orientation of section of skin explants, into which granulocytes with green fluorescence migrated, is unclear. The figures should be clarified by indicating the areas of the epidermis and the dermis by arrows with help of experts of dermatological pathologists.

Response: We apologize for this confusion, this is not indicated in the figure as only the dermis is show, since we have injected the beads in the dermis, and added the neutrophils on the dermis. We have clarified this in the figure legend.

(6) The bottom panel of the Figure 5 is not clear, and probably wrong in some places. This event should be initiated by binding of IgA autoantibodies to skin autoantigens, which are missing. Blister formation should be induced, after migration and activation of neutrophils, although the figure shows the blister as the initial event. In addition, the sites in the epidermis for the blisters and neutrophilic accumulation are different between dermatitis herpetiformis/linear IgA bullous dermatosis (subepidermal area) and IgA pemphigus (or, intercellular IgA dermatosis) (intra-epidermal area). This should be also clearly shown. Positive feedback loop of neutrophil activation is not clearly depicted. The areas of the epidermis and the dermis are not indicated. The figures should be clarified by consulting to dermatologists. In addition, the possible mechanism for the induction of blister formation by activated neutrophils may be included into the figure.

Response: Thank you for these valuable suggestions. Based on your comments, we have now included a sequential order of events in Figure 7. Additionally, we have added several explanatory structures and described how the figure relates to several IgA-mediated diseases in the figure legend.

Reviewer: 2

Comments to the Author

Heineke et al generated a panel of specific peptides mimicking the IgA and FcαRI sequences. They demonstrated that one of linear IgA peptides and a FcαRI peptide decrease IgA-mediated neutrophil migration. Using CLIPs technology to increase peptides' half-life, the authors established cyclic IgA peptide that completely blocked migration of neutrophils. Moreover, the authors have developed a cream containing CLIPS peptide and showed that this can penetrate to skin but not systemically open new therapeutic avenues for IgA-mediated blistering skin diseases.

Comments:

1. The authors should address the role of these cyclic peptides on other neutrophil functions such as reactive oxygen species production and phagocytosis.

Response: Thank you very much for the suggestion. We have included now these results which we agree improved our manuscript. We have tested the capability of these peptides to block other IgA-induced functions of neutrophils, and depicted phagocytosis and ROS production in Figure 2. We have added activation to the title because of these results.

2. They must also test the effect of these peptides on neutrophil migration using IgA independent mechanisms such as MCP-1 and IL-8.

European Journal of Immunology

Response: Thank you for this suggestion. We have tested the effect of those peptides on IL-8 induced neutrophil chemotaxis, and found no effect. We have included these data as Supp. Fig. 3.

Second Editorial Decision

05-Jul-2017

Dear Dr. van Egmond,

It is a pleasure to provisionally accept your manuscript entitled "Peptide mimetics of immunoglobulin A (IgA) and FcαRI block IgA-induced neutrophil activation and migration" for publication in the European Journal of Immunology. For final acceptance, please follow the instructions below and return the requested items as soon as possible as we cannot process your manuscript further until all items listed below are dealt with.

Please note that EJI articles are now published online a few days after final acceptance (see Accepted Articles: http://onlinelibrary.wiley.com/journal/10.1002/(ISSN)1521-4141/accepted). The files used for the Accepted Articles are the final files and information supplied by you in Manuscript Central. You should therefore check that all the information (including author names) is correct as changes will NOT be permitted until the proofs stage.

We look forward to hearing from you and thank you for submitting your manuscript to the European Journal of Immunology.

Yours sincerely,
Marta Vuerich

on behalf of
Prof. Iain McInnes

Dr. Marta Vuerich
Editorial Office
European Journal of Immunology
e-mail: ejied@wiley.com
www.eji-journal.eu

European Journal of
Immunology
